# Diagnosis, treatment, surgical practices and review of the literature in rare coagulation factor deficiencies

**DOI:** 10.1186/s13052-024-01806-7

**Published:** 2025-01-05

**Authors:** Hüseyin Avni Solgun

**Affiliations:** Pediatric Hematology and Oncology, SBU Sisli Hamidiye Etfal Training and Research Hospital, Istanbul, Turkey

**Keywords:** Rare factor deficiencies, Bleeding, Diagnosis, Treatment, Prophylaxis, Surgery

## Abstract

**Background:**

Rare bleeding disorders (RBDs) include fibrinogen (Factor I), prothrombin (Factor II), Factor V(FV), combined Factor V and Factor VIII, Factor VII, Factor X, Factor XI, Factor XII, and Factor XIII deficiencies. This group accounts for 3–5% of all factor deficiencies. Different symptoms may occur, ranging from mild or moderate bleeding to serious and life-threatening bleeding, which may not be related to the factor level. This study aimed to evaluate the diagnosis, genetics, treatment, prophylaxis features and surgical experiences of patients those are followed up in our clinic and the review of the literature of rare factor deficiency.

**Methods:**

Demographic data, number of follow-up visits throughout the study period, clinical symptoms, number and locations of bleeding symptoms of 19 patients diagnosed with RBD (fibrinogen, prothrombin, FV, FVII, FX, FXI or FXIII) who were followed up in our pediatric hematology clinic between year 2023–2024 and complications, inhibitor levels, previous operations, treatment and prophylaxis approaches are recorded in the patient chart and all data had been evaluated retrospectively. In our article, all patients included in this study are mentioned according to the consecutive numbering system as Patient 1(P1) to P19 in Table 2. A comprehensive literature search was performed in PubMed and after primary elections 4 studies are selected from total 23 studies those are most relevant to RBDs in pediatric age as there is only plenty of articles about RBDs. Most of the other studies are reviews without clinical patient trails just including recommadations for diagnosis and laboratuary screenings. In contrast, our study includes a clinical trail on diagnosis, treatment and prophylaxis information of 19 patients with RBDs.

**Results:**

The average age of total 19 patients was 11.2 years (range 2,5–17 years). 14 patients were boys (74%) and 5 patients were girls (26%). 10 of the patients (52%) had FVII deficiency (mean FVII: 8,3%, range 2,5–17%), 4 of patients (21%) had FX deficiency (mean FX:16,2%, range 15–17%) and 4 of patients (21%) had FV deficiency (mean FV:14%, range 10–17%) and 1 had FXIII deficiency (1%) respectively. The normal range laboratory reference values for rare blood factor levels in our institute (factor V, VII and X deficencies) is 70–120%. In our study group, 63% (12/19) of our patients were diagnosed over one year of age. Considering all of our cases, skin and soft tissue bleedings are listed as 52% (10/19), intraoral bleedings as 42% (8/19), nose bleedings as 63% (12/19), joint bleedings as 42%(8/19) and santral nerveous system(CNS) bleedings as 15%(3/19). Among the serious bleedings of our cases, joint bleeding 42% (8/19) takes the first place with followed by CNS bleeding 15% (3/19) and gastro-intestinal system(GIS) bleeding (15%) (3/19) respectively. Among totally 19 patients, FX deficiency-P17 had a null mutation of FX gene and FV deficiency-P3 had a missense mutation of FV gene has been detected those both were severe deficencies. The medical genetics of the sibling patients with combined FVII deficency and hypofibrinogenemia have been evaluated, but the genetic results have not been completed yet.

**Conclusions:**

We believe that data-based service is required in every clinic and healthcare system for early diagnosis and follow-up of RBDs. Additionially family screenings and more effective genetic counseling may heal the overall survival and prevent further severe complications. Moreover; the missing factor, severity of deficiency, personal and family history of bleeding or thrombosis, availability of treatment options, plasma half-life of infused exogenous clotting factors and infusion frequency, advantages and disadvantages should all be considered before a prophylaxis program or treatment of RBDs.

## Introduction

Rare bleeding disorders defined as rare diseases or disorders caused by the deficiency of clotting factors in the blood, affecting very few people worldwide. It is a heterogeneous group of coagulation disorders characterized by fibrinogen, prothrombin, factors V, VII, X, XI, XIII (FV, FVII, FX, FXI or FXIII, respectively) and combined factor V /FVIII defieceny. They are usually transmitted as autosomal, recessive disorders, and the prevalence of severe forms can vary from 1 case in 500,000 for FVII to 1 in 2–3 million for FXIII among general population [1].

Prolonged prothrombin time (PT) or activated partial thromboplastin time (aPTT) tests may be found in these patients. Additionally, in FV deficiency, prolonged bleeding time may be observed in approximately 1 in 3 patients. While plasma fibrinogen is too low to be measured in afibrinogenemia, it is 20–50 mg/DL in its mild form called hypofibrinogenemia. In combined FV/FVIII deficiency, both PT and aPTT are prolonged. In a patient with bleeding problems, clotting factor deficiency may be present even PT and APTT tests are normal. If von Willebrand disease tests are found to be normal in these patients, FXIII deficiency should be investigated. Severe FXIII deficiencies can be revealed by the clot lysis test. All these tests should be evaluated separately for each RBDs [2].

Patients affected by RBDs may present with a wide range of clinical symptoms, ranging from mucocutaneous bleeding, common to all types of RBDs, to life-threatening symptoms such as central nervous system and gastrointestinal bleeding even in mild RBDs [2, 3]. This contrast clinical relationship of factor levels and missing factor have displayed in Table 1. Treatment of these disorders is based primarily on replacing the missing factor using specific plasma-derived or recombinant products. In countries where these facilities are not available, bleeding can be managed using cryoprecipitate, fresh frozen plasma (FFP), or virus-inactivated plasma. Minor bleeding can be managed using antifibrinolytic agents [3].

**Table 1:** Clinical relationship of factor levels and missing factor

Factor deficiency	Clinical corralation
Fibrinogen (FI)	Strong
Prothrombin (FII)	Strong
FV	Weak
FV + VIII	Weak
FVII	Weak
FX	Strong
FXI	Very weak
FXIII	Strong
Vit K dependent factors	Weak

Recently, 2 new drugs, recombinant FXIIIA and a plasma-derived FX, have been added to the existing list of specific hemostatic factors, but there is currently no specific product for prothrombin deficiencies and FV deficiencies [4]. New non-replacement therapies that increase thrombin generation through different mechanisms, such as monoclonal antibody anti-tissue factor pathway inhibitor, RNA interference, and bispecific antibody, an FVIIIa mimetic, are being developed for patients with hemophilia and may be useful in the future.

## Material and method

### Patient selection and registration

Demographic data, number of follow-ups throughout the process, clinical symptoms, number and locations of bleeding episodes, inhibitor levels if any, complications and previous operations, treatment and prophylaxis approaches of 19 patients diagnosed with rare factor deficiency (fibrinogen, prothrombin, FV, FVII, FX, FXI or FXIII) who were followed up in our pediatric hematology clinic between 2023 and 2024 are recorded in the patient chart and had been evaluated retrospectively. All patients included in this study are mentioned according to the consecutive numbering system in Table 2 as P1 to P19.

**Table 2:** Demographic and laboratory characteristics of patients with rare factor deficiency

Patient number(Factor deficiency)	Number of patients	Mean age(years)	Gender	aPTT	PT	INR	Serum Level *P*: all patients respectively)	Symptoms	Genetics	Treatment Blood product	Proflaxis	Clinical properties
P1-2 (F1 + *FVII def.)*	2	7(siblings)	2 famale	Abnormal	Abnormal	Abnormal	14,34 mg/dl	Epistaxis	P1,P2: Not resulted yet	Fibrinogen concentrate Cryoprecipitate on demand	No	None
P3-6 (FV def.)	4	14(10–17)	4 male	Abnormal	Abnormal	Abnormal	7, 5, 27,24%	ICH, Epistaxis Gastrointestinal bleeding Hemarthrosis	P4: FV gene mutation	FFP on demand	FFP 10u/kg twice a week	*P4:Brain hemorrhage at the age of 1, circumcision at the age 8 (fv level was 5.3%.
P7-15(FVII def.)	8	8,3(2,5–17)	2 famale, 6 male	Abnormal	Normal	Normal	1.7, 33, 12, 25, 38, 32,27,32%	Hematoma Hemarthrosis Postoperative bleeding ICH Menorrhagia	No	Recombinant clotting factor VIIa (activated eptacog alfa) (novoseven) on demand	P7:Recombinant clotting factor VIIa two times a week	P7: She had severe intra-abdominal hemorrhage 3 years ago.
P16-18(FX def.)	4	16,2(15–17)	1 famale, 2 male	Abnormal	Abnormal	Abnormal	0.2, 1, 18, 34%	ICH Hemarthrosis Easy bruising Menorrhagia Epistaxis Gastrointestinal bleeding	P16:FX gene mutation	aPCC (cofact) on demand	P16: aPCC 100 u/kg twice a week P17: Apcc 100 u/kg per week	P16: Target joint right ankle more than 10 times a year. *P17:Frequent menorrhagia. Apcc (1000 u/kg per week proflaxi, twice 1000–2000 u/kg per week for on demand menorrhagia)
P19(FXIII def.)	1(P19)	5	boy	Normal	Normal	Normal	1%	UCB ICH Recurrent miscarriage	No	Cryoprecipitate on demand	Cryoprecipitate once a week	A surgical operation was performed in the neurosurgery department after a brain hemorrhage 2 years ago.

*P4: Patient 4 with FV defficency (Preoperative 24 h 2 times a day FFP tranfusion initaited and postoperative 48 h later, proflaxi had continued 1 time a day FFP to 1 week. No complications were observed soon), ICH: Intracranial hemorrhage, aPCC: Activated prothrombin complex coagulation concentrate, UCB: Umblical cord bleeding

### Review data

A comprehensive literature search was performed in PubMed using combinations of the keywords: fibrinogen deficiency/disorder, prothrombin/factor II deficiency, factor V deficiency, factor VII deficiency, combined factor V and factor VIII deficiency, factor X deficiency, factor XI deficiency, factor XIII deficiency, vitamin K dependent coagulation factor deficiencies, rare coagulation factor deficiencies, rare bleeding disorders, surgery, clinical manifestation, treatment, management, inhibitor, thrombosis, bleeding and hemorrhage. In addition, references of selected papers were retrieved to find relevant studies. The 4 most important studies in the literature, which evaluate the diagnosis, treatment and surgical practices in RBDs in the pediatric age group and include clinical follow-up as in our study, were selected for the purpose of literature review.

This manuscript contains just descriptive statistics son no statistical analysis have been applied.

## Results

The average age of total 19 patients was 11.2 years (range 2,5–17 years). 14 patients were boys (74%) and 5 patients were girls (26%). 10 of the patients (52%) had FVII deficiency (mean FVII: 8,3%, range 2,5–17%), 4 of patients (21%) had FX deficiency (mean FX:16,2%, range 15–17%) and 4 of patients (21%) had FV deficiency (mean FV:14%, range 10–17%) and 1 had FXIII deficiency (1%) respectively. The normal range laboratory reference values for rare blood factor levels in our institute (factor V, VII and X deficencies) is 70–120%.

The lowest plasma level was 0.2% in FX deficiency-P16. FVII deficency-P7 and P8 were combined with hypofibrinogenemia. The demographic data, factor levels and characteristics of the patients are displayed in Table 2. In this article, all patients are mentioned according to the consecutive numbering system in Table 2 as P1 to P19 respectively.

FV deficiency-P3 was 16 years old male and had no active bleeding symptoms during prophylaxis with fresh frozen plasma twice week for 2 years. Factor V deficiency-P4 was 10 years old male and had been operated for subdural hematoma and received treatment in intensive care unit for about 1 month when he was 4 years old. Plasma level of FV was %5 (referans level range 70–100%) in this patient. While the patient had been receiving regular prophylaxis with fresh frozen plasma(FFP) twice a week, he had no active bleeding symptom for the last year. FV deficiency-P5 was 17 years old male and had 27% of FV plasma level and had no bleeding episodes for last year without prophylaxis. Both of this patients with FV deficicency had a family history of relatives with FV deficiency.

There is totally 10 patients with FVII deficiency (4 famale, 6 male) included to this study. FVII deficiency-P7 had been using recombinant coagulation factor VIIa preparation with dosage of 15–30 mcg/kg twice a week as prophylaxis for the last year. P7 was under regular prophylaxis and come to physician visits every month had no bleeding episodes in the last year. FVII deficiency-P11 was 5 years 6 months old male and had been referred to our institution due to high plasma PT level from the firstline hospital where they applied to have circumcision operation 1 month ago. In laboratuary plasma level of FVII was 24%, PT was 19.7 s (Normal range of laboratory:9,5–14,1 s), aPTT was 34 s (Normal range of laboratory:21–25 s) respectively.

P1 and P2, who had FVII deficiency together with hypofibrinogenemia, were siblings and both were girls, and their fibrinogen levels were detected as 0.8 g per liter (g/l) and 1.4 g/l, respectively (Laboratory normal range: 1.70–4 g/l). l). In pedigree of this patient, the mother also had hypofibrinogenemia and spontaneous abortion in her first pregnancy, and her last measurement value was 1,2 g/l simultaneously. The father’s fibrinogen values were normal. PT and aPTT tests were also found to be prolonged in these patients.

16 years old male with FX deficiency-P16 had a total of 10 times intra-articular bleeding attacks in both ankles at separate times in the last 2 years. Factor X deficiency-P7 is 17 years old girl with FX plasma level is 1% which shows severe defiency. P19 is the only patient of FXIII type I deficiency is male with a FXIII plasma level of 1.25%.

Of our patients 4/19 (21%) genetic screening had been performed. FX deficiency-P17 had a null mutation of FX gene and FV deficiency-P3 had a missense mutation of FV gene has been detected those both were severe deficencies. The medical genetics of the sibling patients with combined FVII deficency and hypofibrinogenemia have been evaluated, but the genetic results have not been complete yet.

Of our study group cases; skin and soft tissue bleedings are listed as 52% (10/19), intraoral bleedings as 42% (8/19), nose bleedings as 63% (12/19), joint bleedings as 42%(8/19) and santral nerveous system(CNS) bleedings as 15%(3/19). Among the serious bleedings of our cases, joint bleeding 42% (8/19) takes the first place with followed by CNS bleeding 15% (3/19) and gastro-intestinal system(GIS) bleeding (15%) (3/19) respectively.

## Discussion

According to the World Hemophilia Federation(WHF), of the RBDs, FXI and FVII deficiencies are more common and account for 37% and 23% of total RBDs respectively. Fibrinogen and FV deficiencies are 10%, FX deficiency is 9% and FXIII deficiency is 6% respectively. Combined FV and FVIII (3%) and FII (2%) deficiencies have been reported as the rarest bleeding disorders [5]. Rare factor deficiencies are seen in 3–5% of all factor deficiencies. The mode of inheritance of RBDs is generally autosomal recessive [6, 7]. Therefore, unlike hemophilia A and B, it can be seen in girls as well as boys.

In Fisgin Tunc and et al. study between years 1999–2009, total 156 patients from 12 pediatric referral centers were included in the study. The most common RBDs were FVII (53/156, 34%), FV (24/156, 15.4%), and FX (23/156), 14.7%) deficiencies respectively. The most common initial finding in the patients were epistaxis, followed by ecchymosis, and gingival bleeding. In our study group the most common RBDs were FVII (10/19, 52%), FX (4/19, 21%) and FV (4/19, 21%) deficiencies respectively which is similar with Fisgin Tunc et al. Study [8].

In Salcıoglu and et al. study conducted at 1990–2013 included 192 RBDs patients, 142 had FVII, 15 had FX, 14 had FXI, 10 had fibrinogen, six had FV, two had FXIII, two had FV R FVIII and one had FII deficiency. In this study; the bleeding prevalence rates of our symptomatic patients are listed as epistaxis 62.5%, skin bleedings 53%, oral cavity bleeding 28.8%, haematomas 18.3%, CNS bleedings 17.3%, haemarthrosis 14.4%, GIS bleedings 3.8%, menorrhagia 2.9%, haematuria 1.9%, bleeding because of operations 1.9% and iliopsoas bleedings 1.9%. CNS bleedings (41%) take the first place among the serious bleedings of our cases, followed by haemarthrosis (36.4%), GIS bleedings (18.1%) and iliopsoas bleedings (4.5%). Prophylaxy was applied to 9/192(0,04%) patients (5 patients with FVII, 2 patients with fibrinogen and 1 each with FV and FX deficiency) [3]. Table 3 displays characteristics of RBDs cited from the review of the literature.

**Table 3:** Characteristics of rare factor deficiencies obtained from the review of the literature

Factor Deficiency	Gene	Inheritance	Clinical manifestations	Therapeutic blood product	Treatment time
FI^16^	FGA FGB FGG	AR-afibrinogenemia AD-dysfibrinogenemia* AD-Hypofibrinogenemia* ADHypodysfibrinogenemia*	ICH UCB Hemarthrosis Menorrhagia Miscarriage Epistaxis	pd-Fibrinogen concentrate (Haemacompletan), Cryoprecipitate, FFP	On-demand
FII^3,16^	F2	AR	Menorrhagia Hematoma Postpartum hemorrhage Epistaxis ICH UCB Miscarriage	aPCC, FFP	On-demand
FV^3,6,8^	F5	AR	Epistaxis Menorrhagia Gastrointestinal bleeding Hemarthrosis Postoperative bleeding	FFP	On-demand
FV + FVIII^3,6,8^	ERGIC-53 MCFD-2	AR	Easy bruising Epistaxis Postoperative bleeding Gingival bleeding	FFP pd-FVIII, RFVIII, DDAVP	On-demand
FVII^3,6,8^	F7	AR	Hematoma Hemarthrosis Postoperative bleeding ICH Menorrhagia	RFVII, pd FVII, FFP	On-demand
FX^3,6^	F10	AR	ICH UCB Easy bruising Menorrhagia Epistaxis Gastrointestinal bleeding	pd-FX concentrate, pd-FIX/FX concentrates, a PCC, FFP	On-demand
FXI^3,6^	F11	AR AD	Postsurgical bleeding Epistaxis Postpartum hemorrhage Gum bleeding Easy bruising Menorrhagia	pd-FXI concentrate, FFP	On-demand
FXIII^6,8^	F13A F13B	AR	UCB ICH Recurrent miscarriage	FFP, Cryoprecipitate, pd-FXIII-A2B2, rFXIII-A2	Prophylaxis
VKCF^6^	VKOR GGCX	AR	ICH UCB Ecchymosis Mucocutaneous bleeding Posttraumatic hemorrhage	FFP, Vitamin-K1 4-factor, aPCC	On-demand

F: Factor, FFP: Fresh frozen plasma, PCC: Prothrombin complex concentrate, pd: Plasma-derived, rFVII: recombinant factor VII, rFXIII-A2: recombinant factor XIII-A2, ERGIC-53: Endoplasmic reticulum golgi intermediate compartment protein 53, Lman-1: lectin mannose binding protein 1, VKOR: vitamin K 2, 3-epoxide reductase, GGCX: γ-glutamyl carboxylase, AD: Autosomal dominant, AR: Autosomal recessive, VKCF: Vitamin K-dependent coagulation factors, ICH: Intracranial hemorrhage, UCB: Umbilical cord bleeding *Mostly autosomal dominant, X ^3-6-8-16^: Reference number

In comprosion to this clinical trail study with large number of patients; in our study skin and soft tissue bleedings are listed as 52% (10/19), intraoral bleedings as 42% (8/19), nose bleedings as 63% (12/19), joint bleedings as 42%(8/19) and CNS bleedings as 15%(3/19). Among the serious bleedings of our cases, joint bleeding 42% (8/19) takes the first place with followed by CNS bleeding 15% (3/19) and GIS bleeding (15%) (3/19) respectively. The prophylaxis was applied 5/19(26%) of our patients. (2 patients with FX, 1 patient each with FV, FVII and FXIII deficiency). Although epistaxis and intraoral bleedings were at the first-line bleeding symptoms with similar rates to Salcıoglu and et al. study, prophylaxis treatment was applied at a much higher rate in the patients in our study. We think that this is the result of the fact that our study have been conducted n more currently in contrast to Salcioglu study and prophylaxis in RBDs has been strongly recommended in recent years, as in other hemophilias.

Hereditary/congenital FV deficiency is very rare and its frequency is 1 in a million. In factor V deficiency, epistaxis, menorrhagia, skin bleeding, mucosal bleeding and postoperative bleeding are frequently observed [9]. Additionally, umbilical cord bleeding and muscle and joint bleeding have also been reported at low FV levels [10] There is no strong relationship between factor level and bleeding phenotype in factor V deficiency as well as in other RBDs [10]. FV deficiency- P4 is 5 years old male patient and had admitted to the pediatric hematology clinic for circumcision surgery. In laborautary testing FV plasma level was 5.3% (Normal range of laboratory: 70–120%). Preoperatively, FFP was transfused with 10 cc/kg twice a day 24 h before operation. Postoperative following 48 h, FFP was continued with 10 cc/kg/day. The patient P4 had slight bleeding from the glans penis suture site in the postoperative 3rd day during the FFP treatment. Tranexamic acid was started 10 mg/kg twice a day. Afterwhile in 8 h his bleeding was stopped. FFP transfusion with once 10 cc/kg/day was continued to postoperative 1 week. The patient had no other any complications during 1 week follow-up was discharged and no prophylactic treatment was required.

As generally reported, factor VII cases account for the largest ratio of RBDs (66.3%) [11]. Consistent with this information, there are 52%(10/19) cases of FVII deficiency in our study group. Additionally, FV and FX deficiencies were also detected in 21%(for each FV and FX deficiency 4/19).

Hypofibrinogenemia was detected in 2 siblings with FVII deficiency- P1 and P2. In this mild form, called moderate hypofibrinogenemia, the fibrinogen level is noted to be between 20 and 50 mg/dl, as in our patients. Although fibrinogen activity is sufficient for diagnosis, in some cases fibrinogen antigen may also need to be measured. The most common bleeding findings are umbilical cord bleeding, central nervous system bleeding, epistaxis, menorrhagia and oral cavity bleeding [12]. However, hemarthrosis and intramuscular hematoma are present in identified cases. To stop bleeding, a fibrinogen amount of > 50 mg/dl is sufficient. Fibrinogen concentrate (Haemocomplettan) available on the market can be used as treatment [13]. In cases where fibrinogen concentrate not available, FFP transfusion may be the following choice. Since the half-life of fibrinogen is 3 days, continue to treatment depends on the severity of bleeding symptoms [14]. Since these patients did not have any complaints other than P1 who had mild nosebleed episodes 3 times in the last year which can be stopped with compression of sponge in less than 15 min, they were followed up clinically without treatment [15]. There is no information in the literature regarding the coexistence of FVII deficiency and hypofibrinogenemia as in our study and the family members were referred to the medical genetics department for genetic screening. The results will be monitored soon.

FVII deficiency-P8 is 5 years old male and admitted to the pediatric hematology service for circumcision surgery was consulted with the pediatric surgeon. In the examinations performed before circumcision, the patient had a PT of 19.1 s (Normal range of laboratory:9,5–14,1 s) and a FVII level of 24%(normal range %70–100) and F VII inhibitor negative. Recombinant clotting factor VIIa (activated eptacog alfa) was given at 90 µg/kg/dosage 1 h before the operation. During postoperative follow-ups, recombinant clotting factor VIIa was given to the patient with the first dosage 75 µg/kg/dose and subsequent dosages with 50 µg/kg every 6 h in 24 h. The patient P8 continued leakage bleeding from the operation suture and then stopped after the 24th hours postoperative hour. In postoperative period the laborautary screening tests for hemoglobuline and other tests were found to be normal therefore he was discharged after the 72nd hour of operation.

FX deficiency-P16 is 16 years old male had a total of 10 intra-articular bleeding episodes in both ankles at separate times in the last 2 years. Before the follow-up period, when he was 3 years old, he was hospitalized in intensive care once for 2 weeks due to intra-abdominal bleeding. Human prothrombin complex concantrate (HPCC = Factor II/VII/IX/X) had been used in the treatment and prophylaxis of active bleeding. The patient is being followed up due to arthropathy in both ankles. While the patient was receiving prophylaxis twice a week, the dose was switched to once a week as there was no bleeding attack for the last 3 months. FX deficiency-P17 is 17 years old famale patient and her plasma FX level was 1% (normal range is 70–100%) which reveals severe deficency. The patient P17 with frequent menorrhagia had been using 1000 u/kg HPCC once a week, and in case of severe menorrhagia, she was using 1000–2000 u/kg HPCP twice a week. This patient’s bleeding episodes were well-controlled with this prophylaxis -treatment regimen and there was no complaint of additional bleeding.

Only patient with factor XIII deficiency P19 had prolonged gingival bleeding and episodes of bleedings were well-controlled with cryoprecipitate and transexamic acid in the last 2 years follow up. Our hospital’s neonatal clinic plays a major role in the diagnosis and treatment of serious bleedings, especially in the newborn and early infancy periods. Existing factor preparations or FFP were used for treatment generally. The supply of FX and FXIII preparations, which are not yet available mostly worldwide, must be ensured.

A missense mutation was detected in the FXIII gene when the genetic screening tests performed during the active bleeding period as recommended [16]. Table 4 displays recommended on demand and prophylactic treatment of rare bleeding disorders in the guiadance of review of the literature.

**Table 4:** Recommended on demand and prophylactic treatment of rare bleeding disorders factorII, factorV, factorVII, and factorX deficiencies

Factor Deficiency	FactorPlasma Half-Life	Recommended Factor Minimum levels	On-DemandTreatment	ProphylacticTreatment
II	3-4d	%10	*FFP15–25mL/kg *PCC(3-For4-F)20–40U/kg	*FFP not preferred for prophylaxis *PCC(3-For4-F)20–40U/kg1time/wk
V	36 h	>%10	*FFP15–25mL/k	*FFP15–20mL/kg2times/wk
VII	4–6 h	>%20	*FFP not preferred *pdFVIIconcentrate 30–40U/kg *RecombinantFVIIa15–30 mg/kg	*FFP10–20mL/kg2times/wk *pdFVIIconcentrate30–40U/kg3times/wk *RecombinantFVIIa15–30 mg/kg3times/wk
X	40–60 h	>%30	FFP10–20mL/kg PCC(3-For4-F)20–40U/kg pd-FX/FIXconcentrate10–20U/kg pdFXconcentrate25U/kg	FFP notpreferredforprophylaxis PCC(3-For4-F)20–40U/kg2times/wk pd-FX/FIXconcentrate10–20U/kg2times/wk pdFXconcentrate25U/kg2times/wk

Abbreviations: 3-F: 3-factor PCC;4-F:4 factor PCC; FFP: fresh frozen plasma; FVIIa: activated factorVII; PCC: prothrombin complex concentrate; pdFVII: plasmaderived FVII concentrate; pdFX: plasma-derived FXconcentrate; pdFX/FIX: plasma-derived FX and FIX concentrate

Early diagnosis poses a problem in our country as it is worldwide [17]. In our study group; 66% (8/12) of our patients were diagnosed over one year of age. This information indicates that the diagnosis of RBDs may be delayed and that RBDs should be kept in mind in children presenting with acute bleeding, even if their bleeding tests are normal. Bleeding profile can differ in RBDs in contrast to classical hemophilia [18]. Considering all of our cases in this study, skin and soft tissue bleedings are listed as 50%, intraoral bleedings as 40%, nose bleedings as 60%, joint bleedings as 50% and CNS bleedings as 20%.

Genetic studies are an important familial screening and diagnosis method for RBDs [19]. However, today it is not possible to perform genetic testing on RBDs in many centers around the world. Hereditary FV deficiency is inherited in an autosomal recessive manner. Mutation in the factor 5 gene (1q24.2) may cause homozygous or heterozygous transmission [20]. Nearly 200 mutations have been identified so far, most of which are missense and nonsense mutations [21]. While heterozygous carriers are asymptomatic, different degrees of bleeding may occur in homozygous or compound heterozygous carriers [22].

In Fisgin Tunc et al. study, molecular diagnosis was performed in only 2 of the 156(0,01%) patients [8]. In contrast; of our patient group moleculer screening performed to 4 of the 19(21%) patients whereas 2 of them yet not resulted. A null mutation was detected in the FX deficiency-P1 patient, and a missense mutation was detected in our patient with FXIII deficiency. Genetic screening studies are in processes for other patients. Data on FVII genetics are very limited. In a study examining 50 patients diagnosed with FVII deficiency, p.His408Gln gene mutation was detected in 38% of the patients [23].

In contrast to lower genetic screening rates in Fısgın et al. study study, in our patient group 4/19 (21%) genetic screening had been performed. This may be due to the fact that our study was conducted in recent years and the availability of genetic tests has increased. However, like many other genetic based studies in RBDs, their clinical relevance is not yet clear. In this respect, increasing genetic studies may be a pioneer in elucidating why factor levels sometimes do not correlate with clinical findings in RBDs.

We believe that prophylaxis is an option that should be considered in patients presenting with serious bleeding. The information we have regarding prophylaxis is limited, short-term, or covers the duration of a surgical intervention. We believe that our patients have been followed up with personalized prophylaxis choices and a low bleeding and complication rate in the last 2 years. Compared to common bleeding diseases, RBDs are not well defined clinically and their treatments have not been adequately determined. These patients faces up with significant clinical problems before and during diagnosis, and serious difficulties in parameters to determine the diagnosis and treatment [24].

## Conclusions

The diagnosis of RBDs must be included in the differential diagnosis in these rare group of patients, whether or not there is a bleeding complaint, prolonged coagulation tests or family history. In RBDs, the bleeding profile may not correlate with the level of factor deficiency. It is important to diagnose these patients early for treatment to prevent serious bleeding. In addition, family screening and effective genetic counseling are vital and will increase the quality of life in this group of patients by preventing major complications.

## Data Availability

Not applicable.
